# Comparing Needle and Surgical Biopsy in Small Peripheral Non‐Small Cell Lung Cancer With Suspected Pleural Invasion: A Propensity Score‐Matched Study

**DOI:** 10.1111/1759-7714.15491

**Published:** 2024-11-18

**Authors:** Sangil Yun, Taeyoung Yun, Ji Hyeon Park, Bubse Na, Samina Park, Hyun Joo Lee, In Kyu Park, Chang Hyun Kang, Young Tae Kim, Kwon Joong Na

**Affiliations:** ^1^ Department of Thoracic and Cardiovascular Surgery, Seoul National University Hospital Seoul National University College of Medicine Seoul Republic of Korea; ^2^ Cancer Research Institute Seoul National University College of Medicine Seoul Republic of Korea

**Keywords:** needle biopsy, non‐small cell lung cancer, pulmonary surgical procedures, recurrence, visceral pleura

## Abstract

**Background:**

This study aimed to compare long‐term clinical outcomes of percutaneous needle biopsy (PCNB) versus surgical biopsy in patients with peripheral, small‐sized clinical stage 1 non‐small cell lung cancer (NSCLC) with computed tomography (CT)‐defined visceral pleural invasion (VPI).

**Methods:**

We retrospectively analyzed patients who underwent surgery for NSCLC with CT‐defined VPI between 2010 and 2017. We excluded patients with non‐peripheral NSCLC, or cancers > 3 cm. Propensity score matching was carried out to adjust for confounding variables. The primary endpoint was ipsilateral pleural recurrence‐free survival, while secondary endpoints included overall survival and recurrence‐free survival.

**Results:**

Of the 1671 patients with peripheral, small‐sized clinical stage 1 NSCLC with CT‐defined VPI, 805 underwent PCNB, and 866 had a surgical biopsy. Propensity score matching assigned 562 patients to each group. Before matching, the PCNB group demonstrated worse baseline characteristics, including older age, higher smoking history, and more adverse pathological findings. After matching, the 5‐year recurrence‐free survival for ipsilateral pleural recurrence (98.6% vs. 96.0%, *p* = 0.002) and overall survival (93.8% vs. 90.2%, *p* = 0.003) were significantly higher in the surgical biopsy group compared with the PCNB group. Multivariable analysis revealed that PCNB significantly increased the risks of all‐cause mortality and various recurrences before and after matching.

**Conclusions:**

Compared with surgery biopsy, PCNB was associated with higher risks of all‐cause mortality and recurrences, including ipsilateral pleural recurrence. PCNB should be considered with caution in cases of peripheral stage 1 NSCLC where CT‐defined VPI is suspected.

## Graphical abstract

We retrospectively compared PCNB and surgical biopsy in peripheral small‐sized NSCLC with CT‐defined VPI using propensity score matching. PCNB was associated with higher risks of all‐cause mortality and various recurrences, including ipsilateral pleural recurrence. These findings suggest PCNB should be cautiously used in patients with suspected pleural invasion.

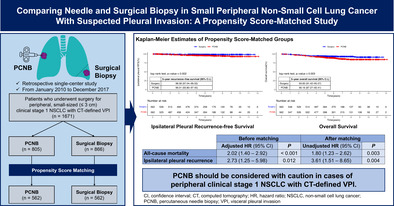

**Abbreviations**
CI: confidence interval
CT: computed tomography
HR: hazard ratio
NSCLC: non‐small cell lung cancer
PCNB: percutaneous needle biopsy
TNM: tumor node metastasis
VPI: visceral pleural invasion

## 1 Introduction

Widespread implementation of lung cancer screening has resulted in an increasing number of early‐stage lung cancer diagnoses, leading to a corresponding increase in surgical treatments [1]. Due to the cost, time, and risks associated with preoperative biopsy, the most recent guidelines strongly recommend surgical biopsy in cases where stage IA non‐small cell lung cancer (NSCLC) is highly suspected [2]. Advances in diagnostic accuracy have shown that following surgical biopsy, over 90% of pulmonary nodules highly suspected to be lung cancer on computed tomography (CT) are confirmed to be cancer [3].

Percutaneous needle biopsy (PCNB) is a pivotal method of confirming the diagnosis of peripherally located early‐stage NSCLC. Due to its minimally invasive nature, PCNB has become increasingly popular, with advancements in imaging technologies enhancing its accuracy and safety [4]. However, debates about potential seeding and ipsilateral pleural recurrence persist [5]. A previous study suggested that CT‐guided PCNB might increase pleural implantation risk, especially pathologically stage 1B cases with subpleural lesions [6]. However, another retrospective study of 321 patients with stage 1 lung cancer discovered that only one of the eight ipsilateral pleural recurrences occurred in a patient who underwent PCNB, arguing that PCNB is not a significant risk factor for pleural recurrence [7].

Clinically, violating the visceral pleura by PCNB before surgery is inferred to significantly increase the likelihood of direct exposure of cancer cells to the pleural space, especially in patients suspected of visceral pleural invasion (VPI). However, to date, no research has been conducted on whether PCNB or surgical biopsy for NSCLC with suspected pleural involvement on chest CT affects long‐term clinical outcomes, such as ipsilateral pleural recurrence and overall survival after surgery. This study aimed to evaluate the comparative clinical outcomes of PCNB versus surgical biopsy, specifically in the context of ipsilateral pleural recurrence in peripheral, small‐sized NSCLC with CT‐defined VPI.

## 2 Methods

### 2.1 Ethics Statement

The study protocol was reviewed by the Institutional Review Board and approved as a minimal‐risk retrospective study (approval date: 27/12/2023, approval number: H‐2312‐074‐1492), and individual consent was waived.

### 2.2 Study Population

**FIGURE 1:**
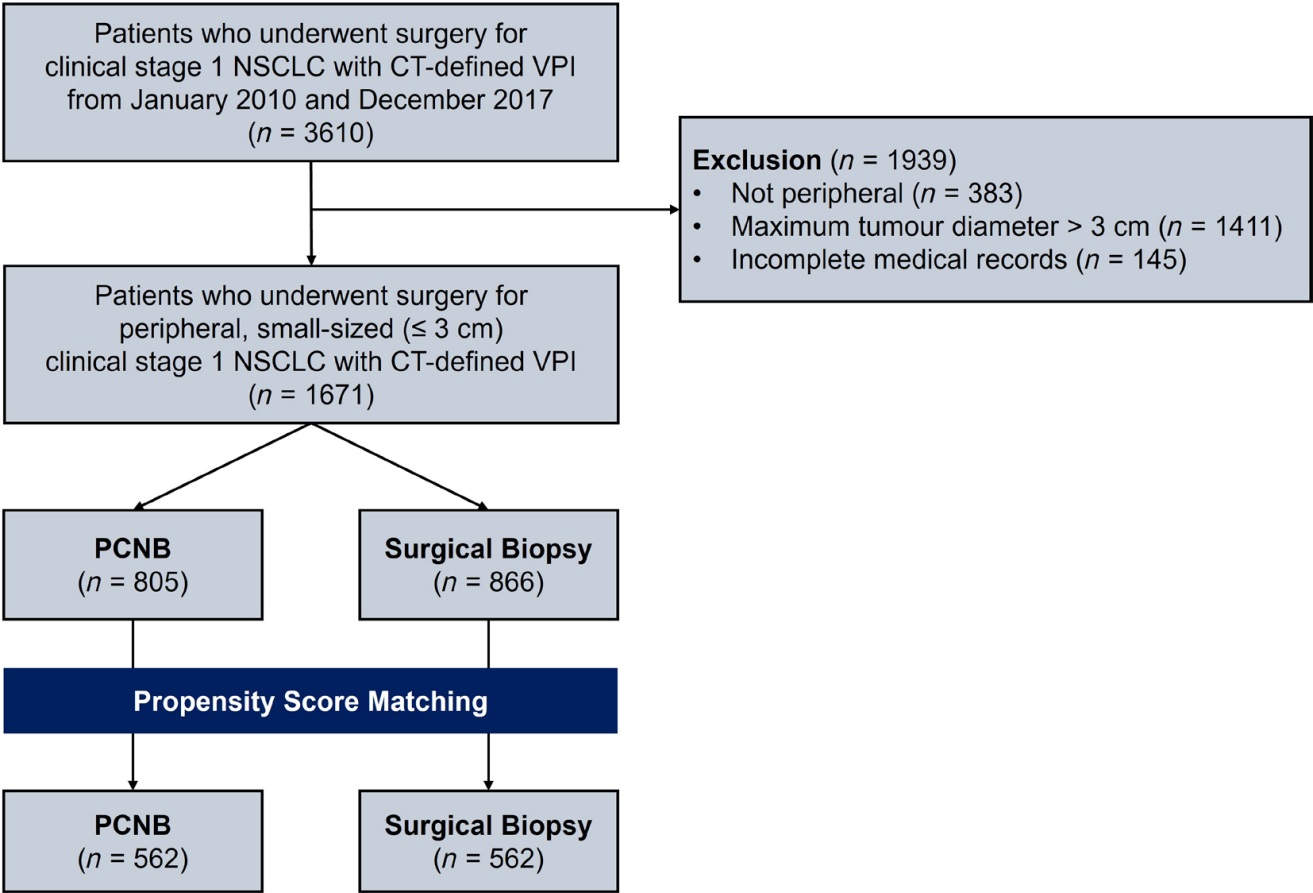
Flow diagram of patient enrollment. CT, computed tomography; NSCLC, non‐small cell lung cancer; PCNB, percutaneous needle biopsy; VPI, visceral pleural invasion.

From January 2010 to December 2017, 3610 patients underwent surgery for clinical stage 1 NSCLC with CT‐defined VPI. After excluding patients with non‐peripheral NSCLC (*n* = 383), tumors with a maximum diameter of ≥ 3 cm (*n* = 1411), and insufficient medical records (*n* = 145), 1671 patients were enrolled in the study (Figure 1).

### 2.3 Radiological Evaluation of Tumor

Peripherally located pulmonary nodules were defined as those located in the outer one‐third of the lung on thin‐section CT [8]. CT‐defined VPI was defined by direct tumor contact with the pleura [9], presence of pleural retraction [9], tag [10], and thickening [11]. All tumors were staged according to the eighth edition of the tumor node metastasis (TNM) classification of malignant tumors [12].

### 2.4 Procedures

The thoracic radiologists at our institution performed PCNB according to the 2020 Korean Society of Thoracic Radiology guideline for PCNB [13]. If NSCLC was confirmed on PCNB, surgery was performed for curative purposes. In cases where lung cancer was suspected radiologically but was not confirmed pathologically on PCNB or proved to be sub‐diagnostic, the decision to perform surgery was made following a multidisciplinary discussion. If NSCLC was not confirmed before curative surgery, a surgical biopsy was performed intraoperatively, and if cancer was confirmed on the frozen section, curative resection was performed. Surgical biopsy refers to biopsy‐purpose surgical resections, such as wedge resection or segmentectomy. In instances where lung cancer was not confirmed on PCNB, the case was classified under the PCNB group rather than the surgery group because the violation of the visceral pleura had occurred due to PCNB before surgery. Consequently, patients who underwent PCNB at any point prior to curative surgery were classified into the PCNB group, while those who did not were classified into the surgery group. Regarding the extent of curative resection, we classified wedge resection or segmentectomy as sublobar resection and lobectomy or bilobectomy as lobar resection.

### 2.5 Outcomes

The primary endpoint was ipsilateral pleural recurrence‐free survival. Secondary endpoints included overall survival, and recurrence‐free survival of other types of recurrences (pleural, locoregional, distant, and overall). We defined overall survival as the time from surgical resection of lung cancer to death from any cause. Recurrence‐free survival was defined as the time from surgical resection of lung cancer to the radiologic or pathologic confirmation of recurrence. Regardless of the side of surgery, a growing pleural lesion on CT or the confirmation of malignant pleural effusion by cytology indicated pleural recurrence [6]. Ipsilateral pleural recurrence was pleural recurrence occurring on the side of the surgery. Locoregional recurrence occurred at the resection margin of the lung, bronchus, hilar or mediastinal lymph node on the side of the surgery [8]. All other types of recurrence were defined as distant recurrence. Any of these types of recurrence were classified as overall recurrence.

### 2.6 Statistical Analysis

Statistical analysis was performed using SAS software, version 9.4 (SAS Institute, Cary, NC), R, version 4.2.2 (R Foundation for Statistical Computing, Vienna, Austria), and IBM SPSS, version 28.0 (IBM Corp., Armonk, NY, USA). Continuous variables are presented as the mean ± standard deviation, while categorical variables are presented as the number and percentage of the subjects. Depending on the distribution of expected frequencies, baseline characteristics were compared using the Chi‐square test or Fisher's exact test for categorical variables. In contrast, for continuous variables, the independent *t*‐test or Wilcoxon rank sum test was used depending on the assumption of normality. The logistic regression was used to calculate estimates using variables considered for matching (age, sex, Eastern Cooperative Oncology Group Performance Score, smoking history, extent of resection, histology, pathologic VPI, pathologic lymphatic invasion, and pathologic node‐positive) as independent variables. Propensity score matching was performed using these estimates, employing the nearest neighbor matching method to ensure the slightest difference in propensity scores between matched pairs. After matching, the comparison of variables considered for matching between groups was assessed using the paired *t*‐test for continuous variables and the McNemar test or Symmetry test for categorical variables. We evaluated the goodness‐of‐fit for the propensity score model using the Hosmer‐Lemeshow test (*C*‐statistic, 0.733; *p* = 0.260; Figure S1). Multivariable Cox regression analysis, with the variables considered for matching as covariates to calculate adjusted hazard ratios (HR), and unadjusted HRs, calculated using multivariable Cox regression while treating each matched dataset as a cluster and accounting for the clustering effect, were used to analyze risk factors for all‐cause mortality. For recurrence, risk factors were analyzed using the Fine‐Gray subdistribution hazard model, considering death as a competing risk, and applying the same approach as for all‐cause mortality. To control for multicollinearity among risk factors during the multivariable risk factor analysis, stepwise selection (entry criterion *p* < 0.050, removal criterion *p* > 0.100) was applied, and only variables significant (*p* < 0.050) were included in the final model. For variables where standard Cox regression could not provide valid estimates, HRs were estimated using Firth's penalized maximum likelihood, with confidence intervals (CI) based on profile penalized likelihood. The significance of each factor was assessed using the penalized likelihood ratio test. All tests were two‐tailed, and a *p* < 0.050 was considered statistically significant. All statistical analyses were conducted with support from the Seoul National University Hospital Medical Research Collaborating Center.

## 3 Results

### 3.1 Baseline Characteristics

Before matching, the PCNB group was older (64.7 ± 10.0 vs. 62.1 ± 9.7, *p* < 0.001) and had fewer women (46.0% vs. 56.1%, *p* < 0.001). The performance score in the PCNB group had fewer cases of 0 (86.7% vs. 91.5%, *p* < 0.001) but more cases of 1 (12.8% vs. 8.1%, *p* < 0.001), and there were fewer never smokers (52.3% vs. 62.6%, *p* < 0.001). There were fewer cases of adenocarcinoma (82.1% vs. 92.6%, *p* < 0.001) but more cases of squamous cell carcinoma (13.2% vs. 4.4%, *p* < 0.001) in the PCNB group. Pathologically, the PCNB group exhibited more T2 stage (33.3% vs. 20.0%, *p* < 0.001), VPI (33.8% vs. 20.8%, *p* < 0.001), lymphatic invasion (17.4% vs. 7.6%, *p* < 0.001), and node‐positive cases (13.0% vs. 3.1%, *p* < 0.001). R1 resection was performed in two patients, both in the PCNB group, and the reason was bronchial resection margin. No complicated resections, such as sleeve lobectomy, were performed (Table 1).

**TABLE 1:** Baseline characteristics before propensity score matching.

Variable	PCNB group (*n* = 805)	Surgery group (*n* = 866)	*p*	SMD
Age	64.7 ± 10.0	62.1 ± 9.7	< 0.001	0.27
Sex, female, *n* (%)	370 (46.0%)	486 (56.1%)	< 0.001	−0.20
ECOG‐PS, *n* (%)			< 0.001	0.21
0	698 (86.7%)	792 (91.5%)		
1	103 (12.8%)	70 (8.1%)		
2	4 (0.5%)	4 (0.5%)		
Smoking history, *n* (%)			< 0.001	0.21
Never smoker	421 (52.3%)	542 (62.6%)		
Ever smoker	384 (47.7%)	324 (37.4%)		
Extent of resection, *n* (%)			< 0.001	0.58
Sublobar resection	89 (11.1%)	298 (34.4%)		
Lobar resection	716 (88.9%)	568 (65.6%)		
Histology, *n* (%)			< 0.001	0.35
Adenocarcinoma	661 (82.1%)	802 (92.6%)		
Squamous cell carcinoma	106 (13.2%)	38 (4.4%)		
Others	38 (4.7%)	26 (3.0%)		
Completeness of resection, *n* (%)			0.142	0.07
R0	803 (99.8%)	866 (100.0%)		
R1	2 (0.2%)	0 (0.0%)		
Pathologic T stage, *n* (%)			< 0.001	0.31
T1	537 (66.7%)	693 (80.0%)		
T2	268 (33.3%)	173 (20.0%)		
Pathologic N stage, *n* (%)			< 0.001	0.46
Not evaluated	14 (1.7%)	68 (7.8%)		
N0	686 (85.2%)	771 (89.0%)		
N1	37 (4.6%)	11 (1.3%)		
N2	67 (8.3%)	16 (1.8%)		
N3	1 (0.1%)	0 (0.0%)		
Pathologic node‐positive, *n* (%)	105 (13.0%)	27 (3.1%)	< 0.001	0.37
Pathologic VPI, *n* (%)	272 (33.8%)	180 (20.8%)	< 0.001	0.30
Pathologic lymphatic invasion, *n* (%)	140 (17.4%)	66 (7.6%)	< 0.001	0.30

*Note*: Continuous variables are presented as the mean ± standard deviation and categorical variables are presented as numbers with percentages.

Abbreviations: ECOG‐PS, Eastern Cooperative Oncology Group Performance Status; PCNB, percutaneous needle biopsy; SMD, standardized mean difference; VPI, visceral pleural invasion.

Following propensity score matching, 1124 individuals were matched, with 562 in each group. All variables had a standardized mean difference within ±0.2, and no statistically significant differences were observed between the two groups (Table 2).

**TABLE 2:** Baseline characteristics after propensity score matching.

Variable	PCNB group (*n* = 562)	Surgery group (*n* = 562)	*p*	SMD
Age	63.4 ± 9.9	63.8 ± 9.1	0.310	−0.04
Sex, female, *n* (%)	280 (49.8%)	299 (53.2%)	0.117	−0.07
ECOG‐PS, *n* (%)			0.968	0.15
0	505 (89.9%)	506 (90.0%)		
1	55 (9.8%)	53 (9.4%)		
2	2 (0.4%)	3 (0.5%)		
Smoking history, *n* (%)			0.094	0.07
Never smoker	313 (55.7%)	332 (59.1%)		
Ever smoker	249 (44.3%)	230 (40.9%)		
Extent of resection, *n* (%)			0.180	−0.05
Sublobar resection	84 (15.0%)	75 (13.4%)		
Lobar resection	478 (85.1%)	487 (86.7%)		
Histology, *n* (%)			0.472	0.04
Adenocarcinoma	498 (88.6%)	504 (89.7%)		
Squamous cell carcinoma	41 (7.3%)	36 (6.4%)		
Others	23 (4.1%)	22 (3.9%)		
Completeness of resection, *n* (%)			0.317	0.06
R0	561 (99.8%)	562 (100.0%)		
R1	1 (0.2%)	0 (0.0%)		
Pathologic T stage, *n* (%)			0.463	0.04
T1	401 (71.4%)	412 (73.3%)		
T2	161 (28.6%)	150 (26.7%)		
Pathologic N stage, *n* (%)			0.422	0.10
Not evaluated	12 (2.1%)	19 (3.4%)		
N0	522 (92.9%)	517 (92.0%)		
N1	7 (1.2%)	10 (1.8%)		
N2	21 (3.7%)	16 (2.8%)		
N3	0 (0.0%)	0 (0.0%)		
Pathologic node‐positive, *n* (%)	28 (5.0%)	26 (4.6%)	0.860	0.02
Pathologic VPI, *n* (%)	164 (29.2%)	154 (27.4%)	0.369	0.04
Pathologic lymphatic invasion, *n* (%)	67 (11.9%)	61 (10.9%)	0.497	0.03

*Note*: Continuous variables are presented as the mean ± standard deviation and categorical variables are presented as numbers with percentages.

Abbreviations: ECOG‐PS, Eastern Cooperative Oncology Group Performance Status; PCNB, percutaneous needle biopsy; SMD, standardized mean difference; VPI, visceral pleural invasion.

### 3.2 Comparison of Overall and Recurrence‐Free Survival Between Propensity Score‐Matched Groups

Before matching, at a median follow‐up of 6.1 years (range 0.0–12.2), all‐cause mortality occurred in 167 cases (10.0%), with ipsilateral pleural recurrence, any pleural recurrence, locoregional recurrence, distant recurrence, and overall recurrence occurring in 45 (2.7%), 47 (2.8%), 172 (10.3%), 165 (9.9%), and 255 (15.3%) cases, respectively. After matching, at a median follow‐up of 6.1 years (range 0.0–12.2), all‐cause mortality occurred in 107 cases (9.5%), with ipsilateral pleural recurrence, any pleural recurrence, locoregional recurrence, distant recurrence, and overall recurrence occurring in 31 (2.8%), 33 (2.9%), 112 (10.0%), 108 (9.6%), and 165 (14.7%) cases, respectively.

**FIGURE 2:**
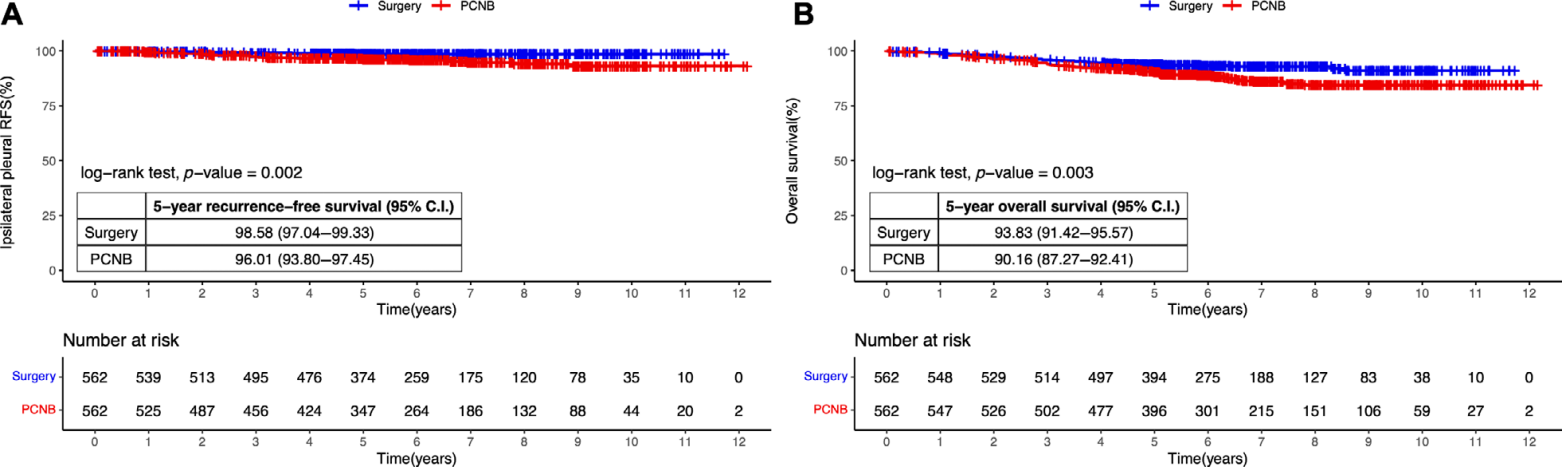
Kaplan–Meier estimates of propensity score matched groups for (A) ipsilateral pleural recurrence‐free survival and (B) overall survival. CI, confidence interval; PCNB, percutaneous needle biopsy; RFS, recurrence‐free survival.

**FIGURE 3:**
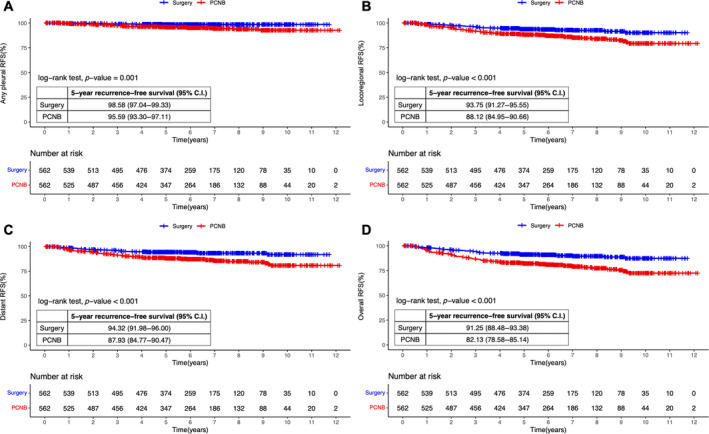
Kaplan–Meier estimates of propensity score matched groups for recurrence‐free survival. (A) Any pleural recurrence. (B) Locoregional recurrence. (C) Distant recurrence. (D) Overall recurrence. CI, confidence interval; PCNB, percutaneous needle biopsy; RFS, recurrence‐free survival.

When comparing the propensity score matched groups, the 5‐year recurrence‐free survival for ipsilateral pleural recurrence was 98.6% (95% CI, 97.0%–99.3%) in the surgery group and 96.0% (95% CI, 93.8%–97.5%) in the PCNB group, and both groups differed significantly (*p* = 0.002) (Figure 2A). The 5‐year overall survival was 93.8% (95% CI, 91.4%–95.6%) in the surgery group and 90.2% (95% CI, 87.3%–92.4%) in the PCNB group, demonstrating a statistically significant difference (*p* = 0.003) (Figure 2B). Compared with the PCNB group, the superior 5‐year recurrence‐free survival in the surgery group was consistently observed in other types of recurrence as well: any pleural recurrence (98.6% vs. 95.6%, *p* = 0.001), locoregional recurrence (93.8% vs. 88.1%, *p* < 0.001), distant recurrence (94.3% vs. 87.9%, *p* < 0.001), and overall recurrence (91.3% vs. 82.1%, *p* < 0.001) (Figure 3).

### 3.3 Risk Factor Analysis for Recurrence and All‐Cause Mortality

In the univariable and multivariable Cox regression analyses conducted before matching, PCNB and pathologic VPI, lymphatic invasion, and node‐positivity were consistently significant risk factors for all‐cause mortality and all types of recurrence (Tables S1–S6).

**FIGURE 4:**
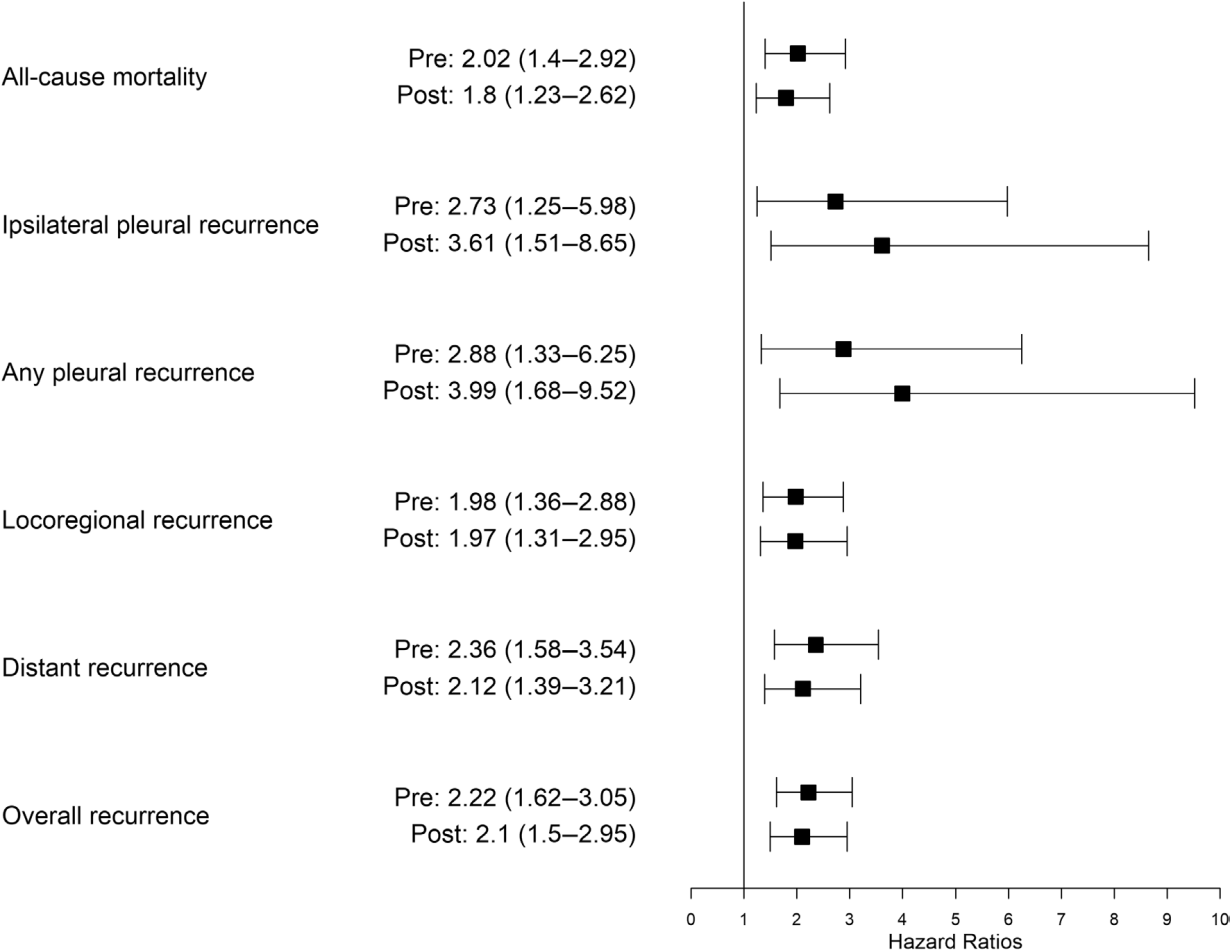
Forest plot of the impact of percutaneous needle biopsy (PCNB) on all‐cause mortality and recurrences. The numbers in the middle column represent the hazard ratios for PCNB corresponding to the events on the left, both before matching (pre) and after matching (post). The numbers in parentheses indicate the 95% confidence intervals.

After adjusting for the variables used in matching, the hazard ratio of ipsilateral pleural recurrence was 2.73 in the PCNB group compared with the surgery group (95% CI 1.25–5.98, *p* = 0.012). Following propensity score matching, risk factor analysis considering the clustering effect of matched data showed that the hazard ratio of ipsilateral pleural recurrence was 3.61 in the PCNB group compared with the surgery group (95% CI 1.51–8.65, *p* = 0.004). Before matching, we observed a trend favoring the surgery group over the PCNB group in all‐cause mortality and other types of recurrences: all‐cause mortality (2.02; 95% CI, 1.40–2.92; *p* < 0.001), any pleural recurrence (2.88; 95% CI, 1.33–6.25; *p* = 0.007), locoregional recurrence (1.98; 95% CI, 1.36–2.88; *p* < 0.001), distant recurrence (2.36; 95% CI, 1.58–3.54; *p* < 0.001), and overall recurrence (2.22; 95% CI, 1.62–3.05; *p* < 0.001). This trend remained consistent after matching: all‐cause mortality (1.80; 95% CI, 1.23–2.62; *p* = 0.003), any pleural recurrence (3.99; 95% CI, 1.68–9.52; *p* = 0.018), locoregional recurrence (1.97; 95% CI, 1.31–2.95; *p* = 0.001), distant recurrence (2.12; 95% CI, 1.39–3.21; *p* < 0.001), and overall recurrence (2.10; 95% CI, 1.50–2.95; *p* < 0.001) (Table 3, Figure 4).

**TABLE 3:** Impact of percutaneous needle biopsy (PCNB) on all‐cause mortality and recurrences.

Event	Before matching		After matching	
	Adjusted HR (95% CI)	*p*	Unadjusted HR (95% CI)	*p*
All‐cause mortality	2.02 (1.40–2.92)	< 0.001	1.80 (1.23–2.62)	0.003
Ipsilateral pleural recurrence	2.73 (1.25–5.98)	0.012	3.61 (1.51–8.65)	0.004
Any pleural recurrence	2.88 (1.33–6.25)	0.007	3.99 (1.68–9.52)	0.018
Locoregional recurrence	1.98 (1.36–2.88)	< 0.001	1.97 (1.31–2.95)	0.001
Distant recurrence	2.36 (1.58–3.54)	< 0.001	2.12 (1.39–3.21)	< 0.001
Overall recurrence	2.22 (1.62–3.05)	< 0.001	2.10 (1.50–2.95)	< 0.001

## 4 Discussion

This study demonstrated two main findings. First, PCNB has significant adverse effect on all‐cause mortality and ipsilateral pleural recurrence, with an adjusted HR of 2.02 and 2.73, respectively, before matching, and an unadjusted HR of 1.80 and 3.61, respectively, after matching. Second, PCNB significantly increased the risk of all‐cause mortality and all types of recurrences, before and after matching.

VPI is associated with poor prognosis in NSCLC and is a T descriptor in the TNM staging system [12]. While some studies suggest that VPI is not a poor prognostic factor in small (≤ 3 cm) lung cancers [14, 15, 16], a meta‐analysis reported a significant association with increased mortality and recurrence in stage I NSCLC patients, indicating that the impact of VPI is independent of tumor size [17]. Previous studies did not specify tumor location. However, our study focused on peripheral, small‐sized NSCLC and found that pathological findings, including VPI, were significant risk factors in both pre‐ and post‐propensity score matching analyses. This underscores the importance of VPI as a prognostic factor, even in small‐sized NSCLC.

PCNB has been widely used for preoperative lung cancer diagnosis. However, owing to the nature of the transpleural approach, this technique has been suspected as a significant risk factor for pleural recurrence, although this remains controversial. Some studies have argued for PCNB innocence, citing that patients who underwent PCNB had fewer ipsilateral pleural recurrences compared with those who underwent bronchoscopic biopsy or wedge resection, although the difference was not statistically significant [7, 18]. Conversely, other studies have shown 8.6% ipsilateral pleural recurrence in the PCNB group compared with 0.9% in the non‐PCNB group, with statistical significance [5]. Furthermore, some research suggests that PCNB significantly increases the risk of pleural recurrence in subpleural early‐stage lung cancer [6, 19], leading to a recommendation against the use of this technique for subpleural lesions [19]. A meta‐analysis conducted to overcome the limitations of small sample sizes in previous studies reported that, compared with other diagnostic methods, PCNB resulted in a higher risk of ipsilateral pleural recurrence with HR of 2.58, as well as a higher risk of other types of metastases with HR of 1.99 [20].

Our results are consistent with recent studies that show that PCNB increases ipsilateral pleural recurrence in subpleural lesions where VPI is suspected. Notably, in our study, PCNB showed a higher risk of ipsilateral pleural recurrence, and all‐cause mortality, as well as other types of recurrence, such as pleural, locoregional, and distant recurrences, compared with surgical biopsy in patients with clinical stage 1 NSCLC with CT‐defined VPI. The increase in ipsilateral pleural recurrence might be explained by the violation of the visceral pleura by PCNB, which could lead to cancer cells spreading to this region. As only NSCLC patients suspected for VPI were included in our study, the chance of cancer cells spreading into pleural space might be higher than in the cohorts of other studies.

Moreover, PCNB increased the risk of all‐cause mortality and other types of recurrences; this might be attributed to multiple factors. Similar to previous studies, pre‐matching analysis demonstrated that pathological findings such as VPI and lymphatic invasion significantly increased locoregional recurrence, which previous studies have corroborated [21, 22]. Nevertheless, after matching, PCNB still increased locoregional recurrence risk. This suggests that tumor violation by PCNB could lead to infiltration of tumor cells into the pleura, stroma, and lymphovascular structures, potentially contributing to locoregional and distant recurrence. Li, Chen, and Zhao [23] conducted a meta‐analysis on the relationship between PCNB and recurrence in stage 1 lung cancer, concluding that PCNB did not significantly increase pleural or total recurrence and that recurrence rates were more closely related to tumor malignancy than the biopsy method. However, in the subgroup analysis of patients with subpleural lesions, PCNB significantly increased pleural recurrence by approximately 11%–25%. The authors speculated that the tumor location and the tumor‐disrupting diagnostic method of PCNB could facilitate microscopic lymphoid infiltration and visceral pleural microinfiltration [23]. Our study explored the possibility that, even in small tumors with or close to VPI, cancer cell infiltration due to PCNB might not be confined to the visceral pleura but may adversely affect locoregional and systemic recurrence. We cannot entirely exclude the influence of variables not included in our matching, such as specific tumor size or genetic profile, and the increase in various types of recurrence is highly likely to have impacted overall survival [23, 24, 25].

This study has some notable limitations. First, the retrospective design and lack of randomization may have introduced inherent biases, such as selection bias and measurement bias. To mitigate this, we employed propensity score matching, although residual uncontrolled biases may remain. Second, the single‐institution nature of the study limits external validation, thereby restricting both reproducibility and generalizability. Further prospective, multi‐center studies are warranted to address these limitations. Third, several variables that could potentially influence the study outcomes were not included in the propensity score matching. For example, we did not account for the experience of the proceduralists or minimally adjust for inter‐proceduralist heterogeneity, nor did we include tumor‐related factors such as tumor grade, size, and location of the biopsied lesion, which may be associated with technical difficulty, subsequent treatment information following recurrence, or resection outcomes (R0/R1), and whether the resection was complicated or uncomplicated. Multiple thoracic radiologists at our institution performed the PCNB procedures, but we did not prospectively collect data on the individual proceduralists, making it impossible to account for their levels of experience. However, as the procedures were performed in accordance with established guidelines [13], we considered that inter‐proceduralist heterogeneity was minimized. Regarding tumor grade, size, and location, we limited our study population to patients who underwent surgery for peripheral, small‐sized (≤ 3 cm) clinical stage 1 NSCLC with CT‐defined VPI, which helped to reduce initial heterogeneity. All patients had pathologic T stage 1 or 2, and among the 441 patients with pathologic T stage 2, only 4 did not have pathologic VPI. Therefore, we considered that including pathologic VPI as a matching variable alone would be sufficient. In fact, although pathologic T stage was not included in the matching process, the groups were well‐matched (Table 2). Information on post‐recurrence treatments was not collected as our focus was on matching patients based on preoperative characteristics and pathologic findings to assess the impact of PCNB on long‐term clinical outcomes. If there were no significant differences in age, sex, ECOG, and pathologic stage, it is likely that there would have been no significant differences in post‐recurrence treatments either. Given the small number of R1 resections, completeness of resection was not included as a matching variable, but the resection outcomes were well‐matched between the PCNB and surgery groups (Table 2). Fourth, segmentectomy and wedge resection were grouped together as sublobar resections. Considering previous meta‐analyses that report better oncological outcomes for segmentectomy compared with wedge resection [26, 27], not distinguishing between these two surgical approaches may affect the interpretation of survival outcomes. Future prospective studies should aim to distinguish between wedge resection and segmentectomy to provide clearer insights into their respective impacts on survival outcomes. Fifth, patients with insufficient medical records were required for the study. These patients represent a small proportion of the total number, but their inclusion could have potentially altered the outcomes.

In conclusion, compared with surgical biopsy, PCNB was associated with higher risks of all‐cause mortality and various types of recurrences, including ipsilateral pleural recurrence for peripheral, small‐sized NSCLC with CT‐defined VPI.

## Author Contributions

All authors had full access to the data in the study and take responsibility for the integrity of the data and the accuracy of the data analysis. Conceptualization: Kwon Joong Na. Data curation: Taeyoung Yun, Ji Hyeon Park, and Bubse Na. Formal analysis: Sangil Yun. Investigation: Sangil Yun and Taeyoung Yun. Methodology: Kwon Joong Na. Project administration: Hyun Joo Lee. Resources: Samina Park, Hyun Joo Lee, In Kyu Park, Chang Hyun Kang, and Young Tae Kim. Software: Sangil Yun. Supervision: Young Tae Kim. Validation: Ji Hyeon Park and Bubse Na. Visualization: Samina Park. Writing – original draft: Sangil Yun. Writing – review and editing: In Kyu Park, Chang Hyun Kang, and Kwon Joong Na.

## Ethics Statement

The study protocol was reviewed by the Institutional Review Board and approved as a minimal‐risk retrospective study (approval date: 27/12/2023, approval number: H‐2312‐074‐1492), and individual consent was waived.

## Conflicts of Interest

The authors declare no conflicts of interest.

## Supporting information


Data S1.

## Data Availability

The data underlying this article will be shared on reasonable request to the corresponding author.
