# Supplementary material for: Comparing Needle and Surgical Biopsy in Small Peripheral Non‐Small Cell Lung Cancer With Suspected Pleural Invasion: A Propensity Score‐Matched Study
Source: Thorac Cancer. 2024 Nov 18;16(1):e15491. doi: 10.1111/1759-7714.15491 (PMC11729920; doi:10.1111/1759-7714.15491)
Supplement: Supplementary file 1 — Data S1. [file TCA-16-e15491-s001.docx]

**Supporting Information**

**TABLE S1. Univariable and multivariable analysis of risk factors for all-cause mortality before propensity score matching.**

|  | All-cause mortality | | Univariable analysis | | Multivariable analysis | |
| --- | --- | --- | --- | --- | --- | --- |
|  | Yes  (n = 167) | No  (n = 1504) | Unadjusted HR  (95% CI) | *P*-value | Adjusted HR  (95% CI) | *P*-value |
| PCNB, n (%) | 121 (15.0 %) | 684 (85.0 %) | 2.95 (2.10 – 4.14) | < 0.001 | 1.88 (1.32 – 2.68) | 0.001 |
| Age | 67.0 ± 9.2 | 62.9 ± 9.9 | 1.05 (1.03 – 1.07) | < 0.001 | 1.04 (1.02 – 1.06) | < 0.001 |
| Sex, female, n (%) | 50 (5.8 %) | 806 (94.2 %) | 0.38 (0.27 – 0.52) | < 0.001 | 0.51 (0.36 – 0.73) | < 0.001 |
| ECOG-PS, n (%) |  |  |  | < 0.001 |  | 0.007 |
| 0 | 136 (9.1 %) | 1354 (90.9 %) | Reference |  | Reference |  |
| 1 | 28 (16.2 %) | 145 (83.8 %) | 1.90 (1.26 – 2.85) | 0.002 | 1.26 (0.83 – 1.91) | 0.284 |
| 2 | 3 (37.5 %) | 5 (62.5 %) | 7.73 (2.46 – 24.32) | 0.001 | 6.34 (1.95 – 20.59) | 0.002 |
| Ever smoker, n (%) | 101 (14.3 %) | 607 (85.7 %) | 2.26 (1.66 – 3.08) | < 0.001 |  |  |
| Lobar resection, n (%) | 127 (9.9 %) | 1157 (90.1 %) | 0.95 (0.67 – 1.36) | 0.778 |  |  |
| Histology, n (%) |  |  |  | < 0.001 |  | < 0.001 |
| Adenocarcinoma | 119 (8.1 %) | 1344 (91.9 %) | Reference |  | Reference |  |
| Squamous cell carcinoma | 34 (23.6 %) | 110 (76.4 %) | 3.45 (2.36 – 5.05) | < 0.001 | 1.81 (1.17 – 2.78) | 0.007 |
| Others | 14 (21.9 %) | 50 (78.1 %) | 3.05 (1.76 – 5.32) | < 0.001 | 2.64 (1.51 – 4.64) | 0.001 |
| Pathologic VPI, n (%) | 65 (14.4 %) | 387 (85.6 %) | 1.82 (1.33 – 2.48) | < 0.001 | 1.44 (1.04 – 1.99) | 0.030 |
| Pathologic lymphatic invasion, n (%) | 35 (17.0 %) | 171 (83.0 %) | 2.15 (1.48 – 3.12) | < 0.001 |  |  |
| Pathologic node-positive, n (%) | 37 (28.0 %) | 95 (72.0 %) | 3.55 (2.47 – 5.12) | < 0.001 | 3.15 (2.13 – 4.65) | < 0.001 |

Continuous variables are presented as the mean ± standard deviation and categorical variables are presented as numbers with percentages.

CI, Confidence interval; ECOG-PS, Eastern Cooperative Oncology Group Performance Status; HR, Hazard ratio; PCNB, Percutaneous needle biopsy; SMD, Standardized mean difference; VPI, Visceral pleural invasion.

**TABLE S2.** Univariable and multivariable analysis of risk factors for ipsilateral pleural recurrence before propensity score matching.

|  | Ipsilateral pleural recurrence | | Univariable analysis | | Multivariable analysis | |
| --- | --- | --- | --- | --- | --- | --- |
|  | Yes  (n = 45) | No  (n = 1626) | Unadjusted HR  (95% CI) | *P*-value | Adjusted HR  (95% CI) | *P*-value |
| PCNB, n (%) | 36 (4.5 %) | 769 (95.5 %) | 4.52 (2.18 – 9.37) | < 0.001 | 2.91 (1.36 – 6.21) | 0.006 |
| Age | 65.5 ± 10.2 | 63.3 ± 9.9 | 1.03 (0.99 – 1.06) | 0.110 |  |  |
| Sex, female, n (%) | 22 (2.6 %) | 834 (97.4 %) | 0.88 (0.49 – 1.57) | 0.660 |  |  |
| ECOG-PS, n (%) |  |  |  | 0.043† |  |  |
| 0 | 36 (2.4 %) | 1454 (97.6 %) | Reference |  |  |  |
| 1 | 9 (5.2 %) | 164 (94.8 %) | 2.40 (1.16 – 4.97) | 0.018† |  |  |
| 2 | 0 (0.0 %) | 8 (100.0 %) | 4.10 (0.24 – 70.38) | 0.330† |  |  |
| Ever smoker, n (%) | 20 (2.8 %) | 688 (97.2 %) | 1.14 (0.63 – 2.05) | 0.670 |  |  |
| Lobar resection, n (%) | 38 (3.0 %) | 1246 (97.0 %) | 1.63 (0.73 – 3.65) | 0.236 |  |  |
| Histology, n (%) |  |  |  | 0.520 |  |  |
| Adenocarcinoma | 42 (2.9 %) | 1421 (97.1 %) | Reference |  |  |  |
| Squamous cell carcinoma | 1 (0.7 %) | 143 (99.3 %) | 0.25 (0.03 – 1.82) | 0.170 |  |  |
| Others | 2 (3.1 %) | 62 (96.9 %) | 1.03 (0.51 – 2.09) | 0.940 |  |  |
| Pathologic VPI, n (%) | 25 (5.5 %) | 427 (94.5 %) | 3.68 (2.04 – 6.61) | < 0.001 | 2.18 (1.16 – 4.11) | 0.016 |
| Pathologic lymphatic invasion, n (%) | 19 (9.2 %) | 187 (90.8 %) | 6.08 (3.38 – 10.9) | < 0.001 | 2.80 (1.39 – 5.65) | 0.004 |
| Pathologic node-positive, n (%) | 16 (12.1 %) | 116 (87.9 %) | 7.45 (4.05 – 13.7) | < 0.001 | 2.81 (1.34 – 5.89) | 0.006 |

Continuous variables are presented as the mean ± standard deviation and categorical variables are presented as numbers with percentages.

CI, Confidence interval; ECOG-PS, Eastern Cooperative Oncology Group Performance Status; HR, Hazard ratio; PCNB, Percutaneous needle biopsy; SMD, Standardized mean difference; VPI, Visceral pleural invasion.

† The hazard ratios, confidence intervals, and significance were estimated using Firth’s penalization, profile penalized likelihood, and penalized likelihood ratio test, respectively.

**TABLE S3. Univariable and multivariable analysis of risk factors for any pleural recurrence before propensity score matching.**

|  | Pleural recurrence | | Univariable analysis | | Multivariable analysis | |
| --- | --- | --- | --- | --- | --- | --- |
|  | Yes  (n = 47) | No  (n = 1624) | Unadjusted HR  (95% CI) | *P*-value | Adjusted HR  (95% CI) | *P*-value |
| PCNB, n (%) | 38 (4.7 %) | 767 (95.3 %) | 4.77 (2.31 – 9.87) | < 0.001 | 3.12 (1.47 – 6.62) | 0.003 |
| Age | 65.9 ± 10.2 | 63.3 ± 9.9 | 1.03 (1.00 – 1.07) | 0.069 |  |  |
| Sex, female, n (%) | 23 (2.7 %) | 833 (97.3 %) | 0.88 (0.50 – 1.56) | 0.660 |  |  |
| ECOG-PS, n (%) |  |  |  | 0.020† |  |  |
| 0 | 37 (2.5 %) | 1453 (97.5 %) | Reference |  |  |  |
| 1 | 10 (5.8 %) | 163 (94.2 %) | 2.59 (1.29 – 5.19) | 0.008† |  |  |
| 2 | 0 (0.0 %) | 8 (100.0 %) | 4.04 (0.24 – 69.20) | 0.335† |  |  |
| Ever smoker, n (%) | 21 (3.0 %) | 687 (97.0 %) | 1.15 (0.65 – 2.04) | 0.640 |  |  |
| Lobar resection, n (%) | 40 (3.1 %) | 1244 (96.9 %) | 1.72 (0.77 – 3.84) | 0.190 |  |  |
| Histology, n (%) |  |  |  | 0.470 |  |  |
| Adenocarcinoma | 44 (3.0 %) | 1419 (97.0 %) | Reference |  |  |  |
| Squamous cell carcinoma | 1 (0.7 %) | 143 (99.3 %) | 0.24 (0.03 – 1.74) | 0.160 |  |  |
| Others | 2 (3.1 %) | 62 (96.9 %) | 1.00 (0.50 – 2.04) | 0.990 |  |  |
| Pathologic VPI, n (%) | 27 (6.0 %) | 425 (94.0 %) | 3.98 (2.23 – 7.08) | < 0.001 | 2.42 (1.30 – 4.52) | 0.006 |
| Pathologic lymphatic invasion, n (%) | 19 (9.2 %) | 187 (90.8 %) | 5.65 (3.17 – 10.10) | < 0.001 | 2.60 (1.31 – 5.13) | 0.006 |
| Pathologic node-positive, n (%) | 16 (12.1 %) | 116 (87.9 %) | 6.98 (3.82 – 12.70 | < 0.001 | 2.63 (1.28 – 5.41) | 0.009 |

Continuous variables are presented as the mean ± standard deviation and categorical variables are presented as numbers with percentages.

CI, Confidence interval; ECOG-PS, Eastern Cooperative Oncology Group Performance Status; HR, Hazard ratio; PCNB, Percutaneous needle biopsy; SMD, Standardized mean difference; VPI, Visceral pleural invasion.

† The hazard ratios, confidence intervals, and significance were estimated using Firth’s penalization, profile penalized likelihood, and penalized likelihood ratio test, respectively.

**TABLE S4. Univariable and multivariate analysis of risk factors for locoregional recurrence before propensity score matching.**

|  | Locoregional recurrence | | Univariable analysis | | Multivariable analysis | |
| --- | --- | --- | --- | --- | --- | --- |
|  | Yes  (n = 172) | No  (n = 1499) | Unadjusted HR  (95% CI) | *P*-value | Adjusted HR  (95% CI) | *P*-value |
| PCNB, n (%) | 123 (15.3 %) | 682 (84.7 %) | 2.88 (2.07 – 4.01) | < 0.001 | 1.94 (1.37 – 2.73) | < 0.001† |
| Age | 64.2 ± 9.7 | 63.2 ± 9.9 | 1.01 (1.00 – 1.03) | 0.079 |  |  |
| Sex, female, n (%) | 72 (8.4 %) | 784 (91.6 %) | 0.65 (0.48 – 0.88) | 0.005 |  |  |
| ECOG-PS, n (%) |  |  |  | 0.003† |  | 0.022† |
| 0 | 142 (9.5 %) | 1348 (90.5 %) | Reference |  | Reference |  |
| 1 | 30 (17.3 %) | 143 (82.7 %) | 2.01 (1.36 – 2.97) | 0.001† | 1.75 (1.18 – 2.59) | 0.006† |
| 2 | 0 (0.0 %) | 8 (100.0 %) | 1.09 (0.07 – 17.68) | 0.954† | 1.34 (0.08 – 22.02) | 0.837† |
| Ever smoker, n (%) | 93 (13.1 %) | 615 (86.9 %) | 1.71 (1.27 – 2.31) | < 0.001 | 1.60 (1.17 – 2.13) | 0.003† |
| Lobar resection, n (%) | 137 (10.7 %) | 1147 (89.3 %) | 1.17 (0.81 – 1.70) | 0.397 |  |  |
| Histology, n (%) |  |  |  | 0.120 |  |  |
| Adenocarcinoma | 143 (9.8 %) | 1320 (90.2 %) | Reference |  |  |  |
| Squamous cell carcinoma | 22 (15.3 %) | 122 (84.7 %) | 1.69 (1.07 – 2.66) | 0.023 |  |  |
| Others | 7 (10.9 %) | 57 (89.1 %) | 1.07 (0.73 – 1.57) | 0.740 |  |  |
| Pathologic VPI, n (%) | 89 (19.7 %) | 363 (80.3 %) | 3.25 (2.41 – 4.39) | < 0.001 | 2.30 (1.69 – 3.14) | < 0.001† |
| Pathologic lymphatic invasion, n (%) | 57 (27.7 %) | 149 (72.3 %) | 4.41 (3.21 – 6.06) | < 0.001 | 2.43 (1.70 – 3.47) | < 0.001† |
| Pathologic node-positive, n (%) | 44 (33.3 %) | 88 (66.7 %) | 5.12 (3.61 – 7.25) | < 0.001 | 2.37 (1.61 – 3.51) | < 0.001† |

Continuous variables are presented as the mean ± standard deviation and categorical variables are presented as numbers with percentages.

CI, Confidence interval; ECOG-PS, Eastern Cooperative Oncology Group Performance Status; HR, Hazard ratio; PCNB, Percutaneous needle biopsy; SMD, Standardized mean difference; VPI, Visceral pleural invasion.

† The hazard ratios, confidence intervals, and significance were estimated using Firth’s penalization, profile penalized likelihood, and penalized likelihood ratio test, respectively.

**TABLE S5. Univariable and multivariable analysis of risk factors for distant recurrence before propensity score matching.**

|  | Distant recurrence | | Univariable analysis | | Multivariable analysis | |
| --- | --- | --- | --- | --- | --- | --- |
|  | Yes  (n = 165) | No  (n = 1506) | Unadjusted HR  (95% CI) | *P*-value | Adjusted HR  (95% CI) | *P*-value |
| PCNB, n (%) | 125 (15.5 %) | 680 (84.5 %) | 3.58 (2.51 – 5.12) | < 0.001 | 2.36 (1.60 – 3.49) | < 0.001 |
| Age | 64.4 ± 9.4 | 63.2 ± 10.0 | 1.02 (1.00 – 1.03) | 0.053 | 1.01 (1.00 – 1.03) | 0.100 |
| Sex, female, n (%) | 69 (8.1 %) | 787 (91.9 %) | 0.65 (0.48 – 0.89) | 0.006 | 0.76 (0.55 – 1.04) | 0.083 |
| ECOG-PS, n (%) |  |  |  | 0.079 |  |  |
| 0 | 141 (9.5 %) | 1349 (90.5 %) | Reference |  |  |  |
| 1 | 23 (13.3 %) | 150 (86.7 %) | 1.46 (0.94 – 2.26) | 0.092 |  |  |
| 2 | 1 (12.5 %) | 7 (87.5 %) | 1.33 (0.47 – 3.78) | 0.590 |  |  |
| Ever smoker, n (%) | 85 (12.0 %) | 623 (88.0 %) | 1.53 (1.13 – 2.07) | 0.006 |  |  |
| Lobar resection, n (%) | 139 (10.8 %) | 1145 (89.2 %) | 1.62 (1.06 – 2.47) | 0.025 |  |  |
| Histology, n (%) |  |  |  | 0.420 |  |  |
| Adenocarcinoma | 141 (9.6 %) | 1322 (90.4 %) | Reference |  |  |  |
| Squamous cell carcinoma | 17 (11.8 %) | 127 (88.2 %) | 1.29 (0.78 – 2.13) | 0.320 |  |  |
| Others | 7 (10.9 %) | 57 (89.1 %) | 1.06 (0.73 – 1.56) | 0.750 |  |  |
| Pathologic VPI, n (%) | 79 (17.5 %) | 373 (82.5 %) | 2.70 (1.99 – 3.66) | < 0.001 | 1.75 (1.26 – 2.44) | < 0.001 |
| Pathologic lymphatic invasion, n (%) | 55 (26.7 %) | 151 (73.3 %) | 4.22 (3.05 – 5.83) | < 0.001 | 2.06 (1.40 – 3.04) | < 0.001 |
| Pathologic node-positive, n (%) | 50 (37.9 %) | 82 (62.1 %) | 6.14 (4.40 – 8.57) | < 0.001 | 3.00 (1.96 – 4.58) | < 0.001 |

Continuous variables are presented as the mean ± standard deviation and categorical variables are presented as numbers with percentages.

CI, Confidence interval; ECOG-PS, Eastern Cooperative Oncology Group Performance Status; HR, Hazard ratio; PCNB, Percutaneous needle biopsy; SMD, Standardized mean difference; VPI, Visceral pleural invasion.

**TABLE S6. Univariable and multivariable analysis of risk factors for overall recurrence before propensity score matching.**

|  | Overall recurrence | | Univariable analysis | | Multivariable analysis | |
| --- | --- | --- | --- | --- | --- | --- |
|  | Yes  (n = 255) | No  (n = 1416) | Unadjusted HR  (95% CI) | *P*-value | Adjusted HR  (95% CI) | *P*-value |
| PCNB, n (%) | 186 (23.1 %) | 619 (76.9 %) | 3.16 (2.40 – 4.17) | < 0.001 | 2.07 (1.54 – 2.79) | < 0.001 |
| Age | 64.4 ± 9.6 | 63.1 ± 10.0 | 1.02 (1.00 – 1.03) | 0.01 | 1.01 (1.00 – 1.03) | 0.023 |
| Sex, female, n (%) | 105 (12.3 %) | 751 (87.7 %) | 0.62 (0.49 – 0.80) | < 0.001 |  |  |
| ECOG-PS, n (%) |  |  |  | 0.031 |  |  |
| 0 | 218 (14.6 %) | 1272 (85.4 %) | Reference |  |  |  |
| 1 | 36 (20.8 %) | 137 (79.2 %) | 1.50 (1.06 – 2.14) | 0.024 |  |  |
| 2 | 1 (12.5 %) | 7 (87.5 %) | 1.07 (0.38 – 3.03) | 0.900 |  |  |
| Ever smoker, n (%) | 136 (19.2 %) | 572 (80.8 %) | 1.68 (1.32 – 2.15) | < 0.001 | 1.47 (1.15 – 1.89) | 0.002 |
| Lobar resection, n (%) | 202 (15.7 %) | 1082 (84.3 %) | 1.15 (0.85 – 1.56) | 0.370 |  |  |
| Histology, n (%) |  |  |  | 0.120 |  |  |
| Adenocarcinoma | 214 (14.6 %) | 1249 (85.4 %) | Reference |  |  |  |
| Squamous cell carcinoma | 31 (21.5 %) | 113 (78.5 %) | 1.60 (1.09 – 2.34) | 0.016 |  |  |
| Others | 10 (15.6 %) | 54 (84.4 %) | 1.04 (0.75 – 1.44) | 0.800 |  |  |
| Pathologic VPI, n (%) | 127 (28.1 %) | 325 (71.9 %) | 3.05 (2.38 – 3.89) | < 0.001 | 2.10 (1.61 – 2.74) | < 0.001 |
| Pathologic lymphatic invasion, n (%) | 80 (38.8 %) | 126 (61.2 %) | 4.05 (3.11 – 5.27) | < 0.001 | 2.08 (1.60 – 2.85) | < 0.001 |
| Pathologic node-positive, n (%) | 69 (52.3 %) | 63 (47.7 %) | 5.76 (4.37 – 7.61) | < 0.001 | 2.81 (2.03 – 3.97) | < 0.001 |

Continuous variables are presented as the mean ± standard deviation and categorical variables are presented as numbers with percentages.

CI, Confidence interval; ECOG-PS, Eastern Cooperative Oncology Group Performance Status; HR, Hazard ratio; PCNB, Percutaneous needle biopsy; SMD, Standardized mean difference; VPI, Visceral pleural invasion.

**FIGURE S1.** Box plot of propensity score distribution across groups


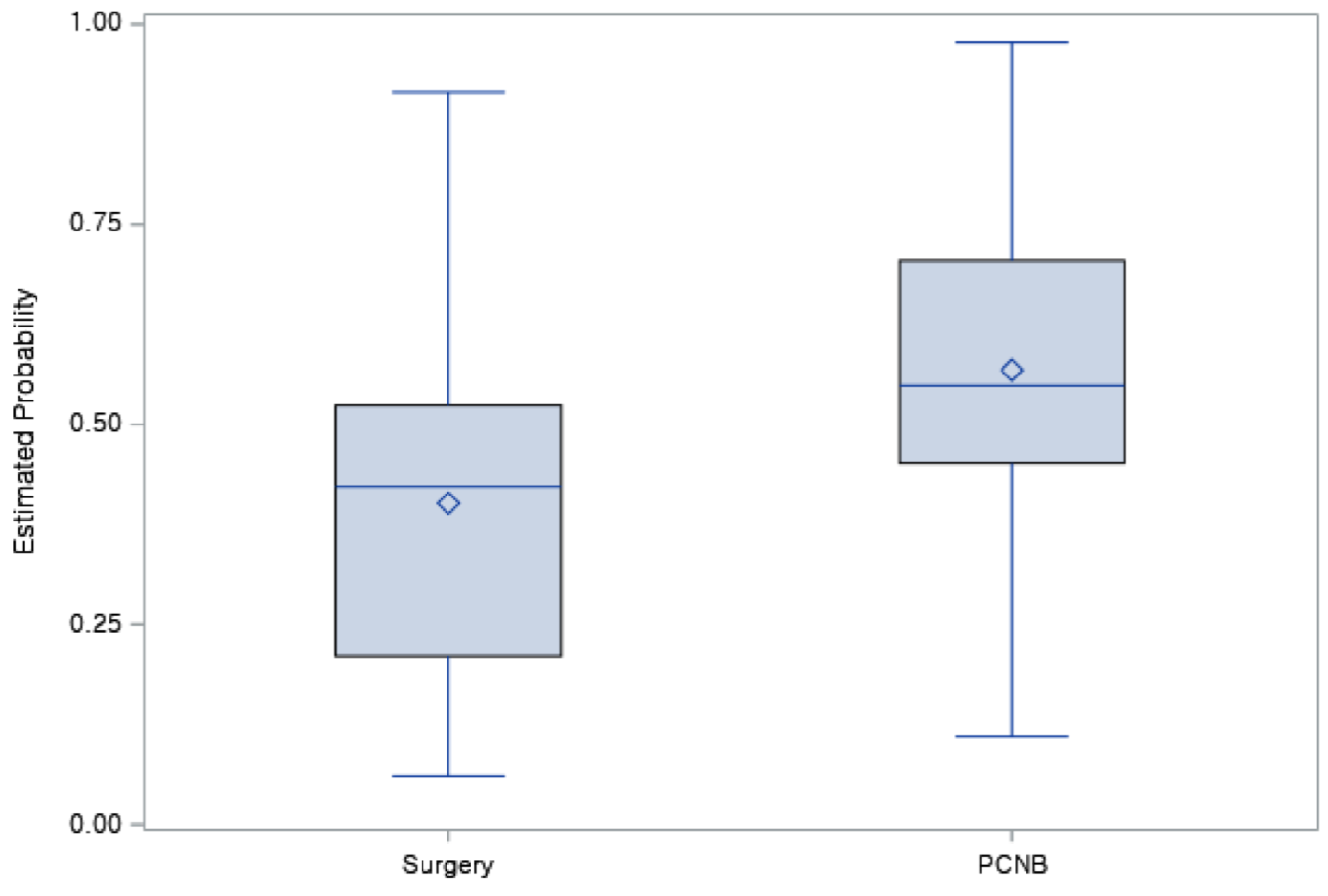


PCNB, Percutaneous needle biopsy
